# Microbial fuel cells for wastewater treatment and electricity production: A multi-platform simulation workflow

**DOI:** 10.1371/journal.pone.0348078

**Published:** 2026-05-07

**Authors:** Muhammad Ali

**Affiliations:** Department of Chemical Engineering, King Fahd University of Petroleum and Minerals, Dhahran, Saudi Arabia; UPES: University of Petroleum and Energy Studies, INDIA

## Abstract

The need for sustainable energy generation and efficient wastewater treatment continues to motivate technologies that integrate energy recovery with environmental remediation. Here, a microbial fuel cell (MFC) system is designed and evaluated, leveraging electrogenic microorganisms to produce electricity during wastewater treatment, with a focus on material selection and design configuration. The proposed design integrates key elements including a proton exchange membrane, anodic and cathodic chambers, and an external circuit to support conversion of organic waste to electrical output through an anode–cathode redox cycle. Four software platforms were used to evaluate complementary aspects of the MFC system under stated modeling assumptions. MATLAB was used to model bacterial growth kinetics and anode performance using Butler–Volmer and Monod based relations. DWSIM was used for thermodynamic screening and mass and energy balance calculations to assess feasibility and stream behavior. COMSOL Multiphysics was used to interpret spatial concentration gradients and biofilm related distributions within the anode compartment. MINITAB was used for a design of experiments style statistical screening (ANOVA and Fisher post hoc testing) of five anode materials using a constructed voltage dataset guided by exploratory prototype observations, and electrode material showed a strong effect on voltage output (F = 21779.35, p < 0.001, R² = 99.83%), with platinum producing the highest mean voltage. Post hoc Fisher testing further indicated that the platinum electrode was statistically different from the other materials. Overall, the study establishes a reproducible simulation workflow that supports comparative screening of anode materials and design configurations for MFC based wastewater treatment with energy recovery.

## 1. Introduction

Conventional wastewater treatment is based on energy intensive aeration, chemical dosing and sludge handling operations. While effective in contaminant removal, these processes can have high operating costs, and do not extract energy from the wastewater’s organic matter. The drive for sustainable and circular solutions has also led to renewed interest in microbial fuel cells (MFCs). MFCs are a bio electrochemical platform for the dual-function of wastewater treatment coupled with electricity generation. Recent review literature has supported this role, with summary of recent progress on key operating principles and performance drivers, as well as specific discussion of growing research on integrated and hybrid wastewater-to-energy configurations, as well as pathways to deployment [1–3].

Hybrid MFC concepts have also been applied for challenging industrial wastewaters. Applications include self-powered MFC–electrolytic cell coupling for the treatment of shale gas flowback wastewater [4], and natural biomass-derived activated carbon anodes for improved electricity output with low-cost anode materials [5]. Economical configurations include dual-chamber salt-bridge MFCs proposed as low-cost and capable of simultaneous wastewater cleaning and electricity generation [6]. Consolidated fermentation MFC systems are capable of large-scale and modular integration in waste-to-energy pipelines and have significant economic advantages [7].

MFCs operate based on the metabolic pathways of electrogenic bacteria. Electrogenic bacteria in wastewater can oxidize organic molecules, releasing electrons and protons as byproducts. Electrons flow through an external circuit to the cathode, generating electrical current, while protons migrate through a membrane and a redox reaction takes place with oxygen at the cathode. This process results in the concurrent removal of organic pollutants and generation of usable electrical power. Recent research has expanded on this, with the evaluation of biological nitrogen removal in MFC-based wastewater treatment [8], and an explanation of how bacterial electron-transport pathways condition electrogenic efficiency [9].

Electrode and interface design have been targeted for performance improvements through carbon-based and composite anodes. Carbon-based and composite anodes include biochar and graphene-based approaches [10,11], with biochar also highlighted for its dual role in pollutant adsorption and energy conversion within MFC operation [12], and carbon composite cathode catalysts in single-chamber systems [13]. Additional enhancement strategies include improving microbial adhesion and electron transfer [14], capacitive-hydrogel bioanodes for improved stack efficiency [15], and electromagnetic wave integration to stimulate microbial activity [16]. Beyond materials, system-level performance depends on operating load and electrical configuration as shown by modeling of external load changes in single-cell and multi-cell series and parallel MFC arrangements [17], and stacked and multi-anodic designs have been proposed to improve scalability and power density [18]. Response surface optimization has also been applied to stacked circulating fluidized-bed MFCs [19].

**Fig 1:**
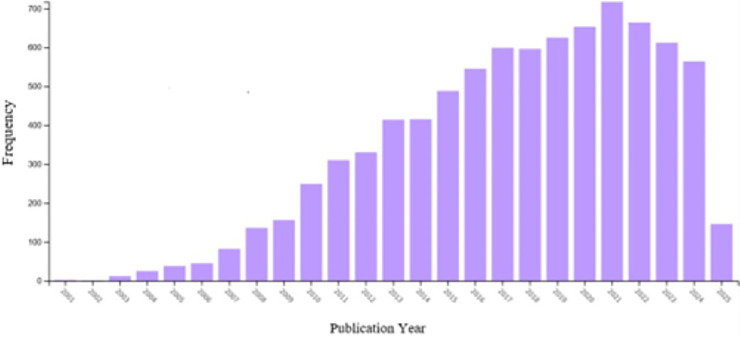
Web of science yearly view of research studies on MFC.

The above-mentioned studies referring to the recent years (2021–2025) highlight the latest research developments in this direction from various aspects. This recent momentum aligns with the broader growth of MFC research output over the past two decades, reflecting sustained interest in bio-electrochemical platforms that link wastewater remediation with energy recovery. As shown in Fig 1, Web of Science publication counts rise steadily from the early 2000s, accelerate markedly around 2010, and remain high in the most recent years, indicating that MFCs have become a prominent research focus within sustainable energy and environmental engineering. This upward trajectory also suggests an expanding scope of investigation, spanning mechanistic studies, materials development, reactor configurations, and scale-up considerations. Notably, the 2025 bar represents a partial-year count.

Although recent MFC studies span mechanisms, electrode and interface design, stacked configurations, and statistical optimization, evaluation efforts are often presented as separate strands. This can obscure how assumptions propagate across analyses and whether screening conclusions are supported consistently by more than one modeling lens. In addition, computational workflows are not always described with enough procedural detail for straightforward reproduction, including clear inputs, boundary conditions, and step-by-step implementation.

This study presents a reproducible, multi-platform simulation workflow for early-stage interpretation and comparative anode-material screening of MFCs under stated assumptions. MATLAB, DWSIM, COMSOL Multiphysics, and MINITAB are used in a complementary sequence to link thermodynamic consistency checks, electrochemical trend modeling, spatial concentration visualization, and statistical screening. The workflow and implementation details are documented through Appendix A and supporting supplementary algorithms and code to enable transparent reuse and reproducibility.

The rest of the study is organized as: Section 2 discusses the typical MFC setup and the equations and principles that govern it; Section 3 models and analyzes different aspects of the MFC using four simulation softwares including MATLAB, DWSIM, COMSOL Multiphysics and MINITAB; Section 4 outlines the environmental, economic and societal benefits of using MFCs; Section 5 concludes the study and provides some future recommendations.

## 2. Microbial fuel cell principles and configuration

An MFC is a bio electrochemical system that links microbial metabolism to electrochemical energy conversion. In an MFC, electrogenic bacteria oxidize organic substrates and release electrons, which are captured and routed through an external circuit to produce electrical power. As a result, MFC configuration and performance depend on how effectively microbial activity, electrode materials, and electrochemical processes are integrated.

### 2.1. System configuration

A typical MFC consists of two chambers:

- **Anode chamber**: Maintained under anaerobic conditions and filled with a liquid medium containing organic matter, this chamber hosts electrogenic bacteria that oxidize the substrates. The anode electrode provides a surface for microbial colonization and direct electron transfer.
- **Cathode chamber**: Maintained under aerobic conditions, the cathode serves as the terminal electron acceptor where oxygen is reduced to water. The two chambers are separated by a **Nafion membrane**, which acts as a proton exchange membrane.

**Fig 2:**
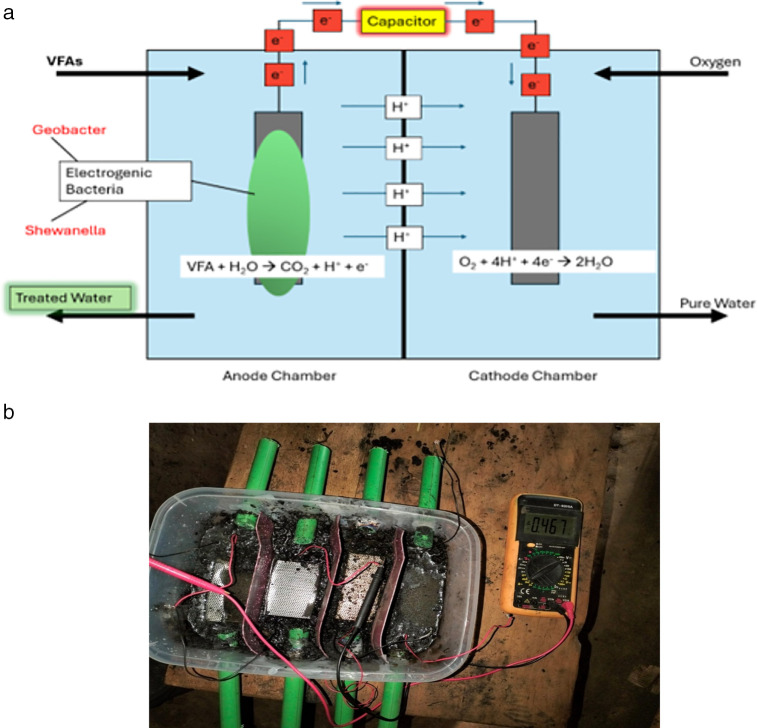
a: Setup of a typical MFC. 2b: Working MFC prototype showing the testing of anode materials.

Electrons travel from the anode to the cathode through an external wire, generating a current, while the protons travel internally through the membrane and combine with electrons and oxygen to form water. Fig 2a shows the schematic diagram of a working MFC (In a later Section 2.3, Fig 2b will present a working MFC prototype).

### 2.2. Reactions and Equations Governing MFCs

This section provides the electrochemical reactions and mathematical equations that are used to set up and optimize MFCs in simulation softwares.

#### 2.2.1. Electrochemical Reactions.

The main redox reactions occurring in an MFC are:

- **Anode (oxidation):** $C_{\text{6}}H_{\text{1}\text{2}}O_{\text{6}}\;+\;\text{6}H_{\text{2}}O\;\to \;\text{6}CO_{\text{2}}\;+\;\text{2}\text{4}H^{+}\;+\;\text{2}\text{4}e^{-}$
- **Cathode (reduction):** $O_{\text{2}}+\;\text{4}H^{+}+\;\text{4}e^{-}\to \;\text{2}H_{\text{2}}O$

- **Overall cell reaction:** $C_{\text{6}}H_{\text{1}\text{2}}O_{\text{6}}\;+\;\text{6}O_{\text{2}}\;\to \;\text{6}CO_{\text{2}}\;+\;\text{6}H_{\text{2}}O$

These reactions are exergonic and provide the energy that can be harvested as electricity.

#### 2.2.2. Governing equations.

The four main governing equations used for modelling the MFC in the simulation softwares are briefly described as follows:

Nernst Equation (Electrode Potential)

To predict how cell voltage changes with concentration and temperature:


$$E\;=\;E^{\circ }-\;\left(\frac{RT}{nF}\right)*ln\;Q$$

where *E* and *E⁰* refer to electrode and standard potentials respectively, *R* refers to gas constant (8.314 J/mol·K), *T* refers to temperature in Kelvin, *n* refers to number of electrons transferred, *F* refers to Faraday constant (96485 C/mol), and *Q* refers to reaction quotient.

### Butler-Volmer Equation (Electrode Kinetics)

This equation models the rate of electron transfer at the electrode surface:


$$i\;=\;i_{\text{0}}\left[exp\left(\frac{\left(\alpha nF\eta \right)}{RT}\;\right)-\;exp\left(\frac{\left(-(\text{1}\;-\;\alpha )nF\eta \right)}{RT}\;\right)\right]$$

where *i* refers to current density, *i₀* refers to exchange current density, *α* refers to charge transfer coefficient, and *η* refers to overpotential, *R* is the universal gas constant, *T* is temperature, and *F* is Faraday’s constant.

In this work, the Butler–Volmer equation is used as a comparative electrochemical framework to illustrate relative trends under consistent modeling assumptions, rather than as a fully predictive kinetic model calibrated to material-specific i₀ and α values.

### Monod Kinetic Equation (Microbial Growth)

Used to model the specific growth rate of bacteria based on substrate concentration:


$$\mu =\;\mu_{max}*\frac{S}{K_{s}+S}$$

where *μ* refers to specific growth rate, *μ*_*max*_ refers to maximum growth rate, *S* refers to substrate concentration, and *K*_*S*_ refers to half-saturation constant.

### Cell Voltage with Losses

The actual voltage output of the MFC accounts for various losses:


$$V_{cell}=\;E_{anode}-\;E_{cathode}-\;\eta_{activation}-\;\eta_{concentration}-\;IR$$

Where *V*_*cell*_ refers to measured cell voltage, *η* terms refer to activation and concentration overpotentials, and *IR* refers to ohmic loss from internal resistance.

### 2.3. Physical MFC Prototype and Exploratory Testing

A laboratory-scale MFC prototype (Fig 2b) was built and tested in this study to confirm basic system feasibility and to support the assumptions used later in the modeling work. The prototype was assembled in an academic laboratory and used as an exploratory platform to observe representative bio electrochemical behavior under practical operating conditions.

The setup used a container-based MFC configuration with an anode and cathode assembly connected through an external circuit. Five electrode materials were qualitatively tested: graphite felt, carbon nanotube, graphene, stainless steel and platinum. Since the experimental data collection was limited by restricted access to microbial cultures, feedstock, and experimental resources, experimental testing was deliberately limited to a few sets of exploratory measurements intended to establish some representative voltage ranges and relative performance trends.

Voltage generation was observed for all electrode materials, which shows that bio electrochemical processes are active in the prototype. These exploratory observations were used to calibrate and inform the generation of simulation-based voltage datasets. In particular, the dataset analyzed in MINITAB was computer-generated to mimic the experimentally informed trends and was used only for comparative statistical assessment. The prototype measurements themselves were not considered to be a statistically rigorous dataset and were not directly used in the statistical analyses presented below.

### 2.4. Multi-Platform simulation workflow

Although Sections 2.1–2.3 establish the operating principles and experimental context, simulation was used to extend the analysis under controlled, repeatable assumptions and to support systematic anode-material comparison when experimental observations are limited. MATLAB was selected to capture time-dependent microbial growth and substrate-consumption trends through Monod-type differential equations. DWSIM was used for steady-state stoichiometric and thermodynamic reaction modeling with flow calculations as a system-level consistency check, noting that species definition and property conditioning are critical when ionic or electron-related terms must be represented indirectly. COMSOL Multiphysics was used to examine spatial transport behavior through PDE-based physics and field-resolved outputs, with manual definition required for certain bio electrochemical species. This combined workflow motivates the model implementations presented in Section 3.

## 3. Modelling MFC using simulation softwares

To comprehensively understand and optimize the electrochemical, biological, and thermodynamic behavior of the MFC system, a combination of simulation tools was utilized using the equation discussed in Section 3. Each software served a distinct role—ranging from modeling chemical reactions and microbial growth to analyzing spatial concentration gradients and validating system performance. This integrated simulation approach enabled a deeper analysis of key design parameters and performance bottlenecks.

### 3.1. DWSIM for thermodynamic representation of MFCs

MFCs are inherently bio-electrochemical systems in which substrate oxidation and electron generation are governed by microbial metabolism, biofilm development, and kinetic limitations. In this study, DWSIM was employed to construct a simplified, steady-state, system-level thermodynamic representation of an MFC-like configuration rather than a mechanistic model of microbial growth or anode kinetics. The RGIBBS reactor was therefore used to impose equilibrium and stoichiometric constraints on overall substrate conversion and product formation, enabling thermodynamic feasibility assessment and comparative trend analysis under idealized assumptions. As such, the DWSIM model does not resolve time-dependent biofilm behavior or microbial reaction rates, and its outputs represent equilibrium-based bounds on glucose conversion and associated species generation rather than predictive biological kinetics.

#### 3.1.1. Simulation setup and model components.

The DWSIM simulation was organized to model the step-by-step movement of substrate through the primary functional units of the MFC system. The simulation framework features four fundamental parts as follows:

- **Feed Stream:** It adds glucose-based organic substrate to the system which represents municipal wastewater containing high biodegradable content.
- **RGIBBS Reactor:** It represents the anode chamber as an equilibrium-based overall substrate-conversion block, used to estimate outlet species distribution under idealized stoichiometric and thermodynamic constraints.
- **Separator Unit:** It functions as a PEM which enables protons to move selectively while preventing the anode and cathode chambers from mixing.
- **Cathode Reactor:** It reduces oxygen by utilizing incoming protons and electrons to complete the electrochemical circuit which results in water formation as a byproduct.

**Fig 3:**
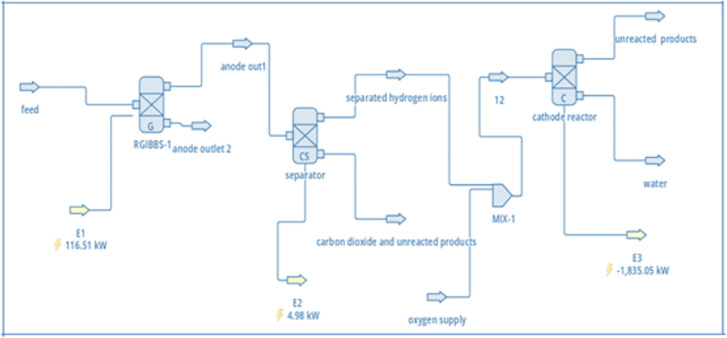
The Model for MFC Simulated in DWSIM.

**Fig 4:**
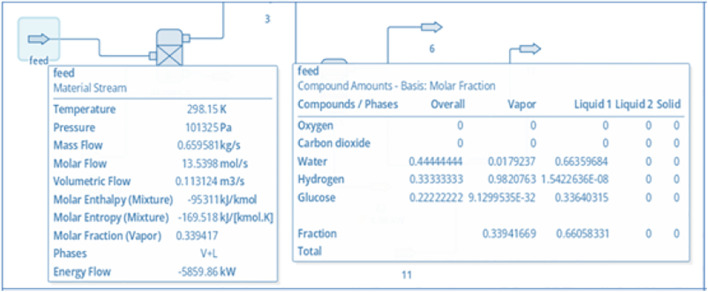
The Feed Properties of MFC Simulated in DWSIM.

The linkage between these components provides a simplified process-flow analogue of an MFC (feed → overall anode conversion → proton-selective separation → cathode reduction) for steady-state mass and energy accounting, rather than a mechanistic representation of microbial electrochemistry. The blocks were specified through stoichiometric reactions as well as component properties and thermodynamic packages tailored to aqueous biochemical systems. Fig 3 shows the complete simulation layout. The feed stream component properties appear in Fig 4 which depicts essential properties including flowrate, temperature, pressure, and molar composition of glucose as well as related species.

#### 3.1.2. Stream Properties.

The RGIBBS reactor and separator unit outlet streams were examined to understand how individual components moved through the MFC system. The analysis focused on how glucose transformed at the anode while tracking hydrogen ion transfer through the PEM and monitoring cathode byproducts.

**Fig 5:**
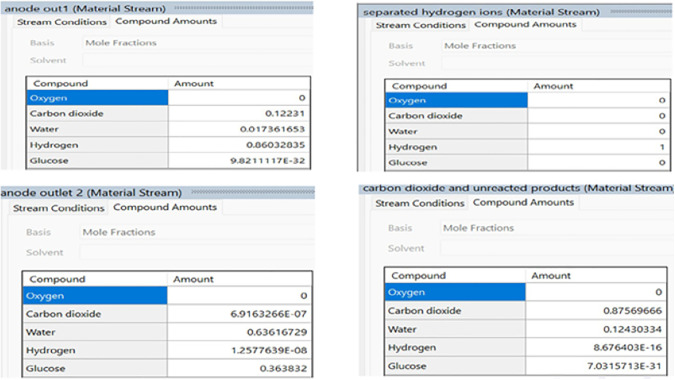
Results Images for Anode and Separator Streams.

The analysis of the anode and separator outlet streams along with separated hydrogen ions, carbon dioxide and unreacted products is given in Fig 5 (which refer to DWSIM images of some selective cases). These results demonstrated a substantial glucose depletion, consistent with the imposed overall conversion assumptions in the equilibrium reactor and the selected reaction constraints used to represent anode-side conversion. Protons formed the largest component of the stream composition along with carbon dioxide and trace amounts of residual glucose and intermediate volatile compounds.

The RGIBBS reactor provided an equilibrium-based estimate of outlet composition for the specified overall conversion case, enabling a thermodynamic bounding analysis of the anode-side products under idealized assumptions. The Separator Outlet Stream analysis showed a distinct proton-dominated composition while showing minimal back diffusion from other molecular species. The selective ionic transport capability of the PEM resulted in this behavior which prevented significant cross-contamination between the anode and cathode chambers.

Analyzing these outlet compositions validated basic bio-electrochemical flow patterns while revealing information about the efficiency of reaction completion and transfer processes. The results of this study established the foundation for analyzing cathodic reactions in subsequent sections. The DWSIM calculations are used as a thermodynamic bounding layer for the same operating envelope examined in the MATLAB and COMSOL analyses, and are not treated as a kinetic representation of bio electrochemical rate behavior. A reproducible workflow for configuring and executing the DWSIM model is provided in Appendix A.

### 3.2. MATLAB for kinetic and electrochemical behavior

To extend the thermodynamic simulation results from DWSIM, we performed dynamic modeling in MATLAB to study microbial kinetics and the electrochemical behavior of MFC system. The simulations enabled us to observe dynamic patterns of substrate utilization alongside biomass development and electrode potential changes in response to different materials. Two primary frameworks guided the development of MATLAB models as listed below:

- **Monod Kinetic Equation**: This provides a framework to model both microbial growth and substrate degradation.
- **Butler-Volmer Equation:** This equation analyzes how various anode materials perform electrochemically at different current densities.

The simulations explored the conditions that deliver both maximum substrate conversion and efficient power generation. The study findings present voltage-current relationships along with electrode material performance comparisons. The following subsections present details about each simulation module.

#### 3.2.1. Anode Material Performance Comparison.

**Fig 6:**
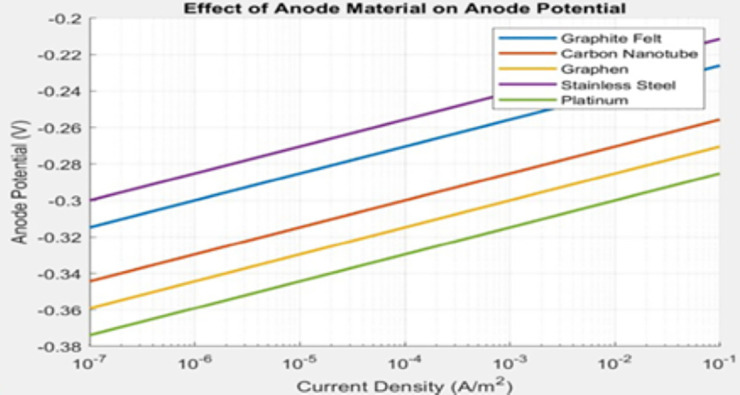
Effect of Anode Material on Anode Potential.

An MFC’s efficiency relies on electrochemical reactions occurring at the anode. MATLAB simulations based on the Butler-Volmer equation tested five anode materials namely Platinum, Graphene, Graphite Felt, Stainless Steel and Carbon Nanotube. The electrode potential depends on current density through this equation which models both anodic and cathodic reaction kinetics. Using the Butler–Volmer model, the MATLAB simulation carried out the same set of representative electrochemical assumptions to create a polarization behavior for each of the five anode materials under controlled variation of current. In Fig 6, the electrode potential is plotted as a function of current density, as simulated for each material. As was observed in the experiments, platinum required the least overpotential and had the best kinetics, followed by graphene and carbon nanotube. The commercial materials, graphite felt and stainless steel, resulted in a larger activation loss than other materials.

Variations in anodic output between these materials is mainly due to the intrinsic properties of the material itself. Electrical conductivity is a factor, as the high conductivity of platinum is able to maintain high electron transport from the biofilm to the external circuit and can decrease activation-related losses. Surface area is also a factor in the attachment of microbes and development of biofilm, as high–specific surface area materials such as graphene and carbon nanotubes can enable denser colonization and higher electron-transfer rates. Finally, surface chemistry and biocompatibility can alter the interface between microbes and electrodes, as certain hydrophilic functional groups and proper surface energy can improve attachment and electron transfer.

To contextualize the MATLAB-based voltage trends, Table 1 summarizes the mean voltage values obtained in this study for each anode material alongside representative literature values. This comparison provides benchmarking context for the magnitude of the simulated outputs under the stated assumptions.

**Table 1:** Comparison of mean voltage values from MATLAB with literature voltage values.

Anode material	Mean voltage in this study (V)	Literature voltage values (V)
Platinum	0.375	0.3 - 0.65
Graphene	0.36	0.4
Graphite felt	0.32	0.3 - 0.4
Carbon nanotube (CNT)	0.342	0.5
Stainless steel	0.3	0.3 - 0.5

Overall, the voltage magnitudes fall within the range commonly reported for comparable MFC setups, with platinum remaining the highest-performing material in both the present study and the literature. Minor differences between the values reported here and literature benchmarks are expected because published studies report voltage under different substrates, external loads, and reactor geometries.

#### 3.2.2. Microbial Growth Dynamics.

MFC performance is influenced not only by electrode properties but also by microbial activity within the anode chamber. In MATLAB, the behavior of electroactive microbes and substrate consumption was simulated using Monod-type differential equations. This formulation represents an idealized Monod growth case with constant kinetic parameters and does not explicitly capture pH evolution or inhibition effects. For this reason, the results are interpreted as baseline trends in biomass growth and substrate depletion under the assumed kinetics.

**Fig 7:**
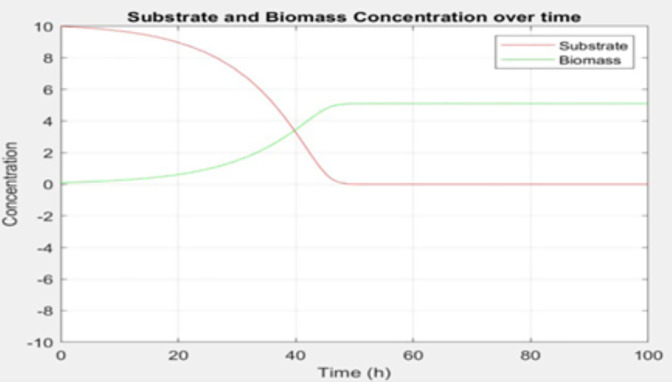
Substrate and Biomass Concentration over Time.

As shown in Fig 7, the biomass profile exhibits an initial lag phase followed by exponential growth and eventual saturation, whereas the substrate concentration decreases rapidly at early times and then approaches a near-zero plateau. Together, these profiles illustrate the expected dynamic coupling between microbial growth and substrate breakdown under Monod kinetics in this simplified representation.

Furthermore, the patterns demonstrated in Fig 7 present a vital question about optimizing the MFC operational timeframe by comparing the benefits of short versus long substrate degradation periods for improved system performance. Fig 7 demonstrates important trends that enable better operational management of the MFC system. Shorter substrate degradation periods are usually advantageous as they demonstrate faster wastewater processing cycles and accelerated electricity production. When substrate consumption speeds up the reactor achieves higher turnover rates which allows for smaller reactor designs and boosts overall system productivity. The rapid use of substrates may result in microbial biofilm development that is insufficient or power outputs that fluctuate over time. Extended operational periods permit complete microbial presence and biofilm stability but might lead to substrate exhaustion before achieving maximum electrical output hence diminishing coulombic efficiency. The polarization and trend outputs from MATLAB define the comparative operating points and current density ranges that are then interpreted spatially in COMSOL and summarized statistically in MINITAB. A reproducible workflow for the MATLAB-based modules, including required inputs and execution sequence, is provided in Appendix A.

### 3.3. COMSOL Multiphysics for spatial transport and concentration distribution

A COMSOL Multiphysics simulation was implemented to qualitatively examine spatial transport behavior and concentration distribution patterns within an MFC-inspired anode domain. A two-dimensional rectangular cross-section was used as a simplified representation of the MFC domain to enable qualitative visualization of concentration and potential gradients. The model therefore focuses on diffusion-driven transport within the section and does not explicitly resolve three-dimensional geometry or flow-field effects. The model focuses on illustrating relative gradients and transport trends associated with substrate availability and electrochemical reaction zones. The biofilm was represented as a static layer adjacent to the anode surface, and representative transport conditions were applied to enable numerical convergence and visualization. As such, the COMSOL model is intended to provide insight into transport-related behavior under simplified assumptions, rather than to resolve microbial growth kinetics or quantitatively predictive biological processes.

#### 3.3.1. Biofilm Behavior in the Anode.

**Fig 8:**
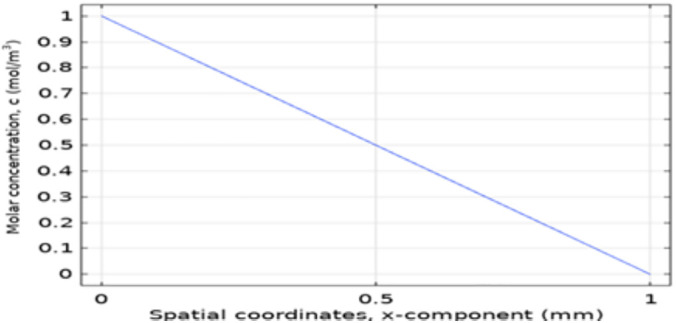
Variation of Biofilm across the Anode Compartment.

The simulation represented microorganisms as a biofilm layer located adjacent to the anode surface, with spatial variation extending outward into the bulk domain. Fig 8 illustrates the spatial distribution of the biofilm layer within the anode compartment as represented in the simulation domain.

The key observations are listed as:

- Higher relative biofilm density was observed near the anode region, consistent with increased local glucose availability and the modeled electrochemical reaction zone.
- Microbial density exhibited a spatial gradient extending from the anode surface into the bulk fluid region.

These spatial trends highlight regions of enhanced electrochemical interaction and substrate availability within the modeled anode domain.

#### 3.3.2. Component Concentration Over Time.

**Fig 9:**
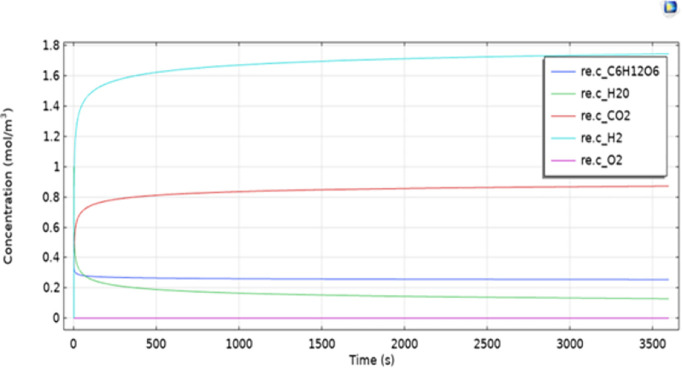
Concentration of Reactants Over Time.

The distribution of glucose, hydrogen ions (H⁺), carbon dioxide (CO₂), and oxygen (O₂) in the domain was simulated using COMSOL. The concentration profiles shown in Fig 9 indicate the temporal evolution of key chemical species within the modeled domain.

The following trends were observed:

- Glucose concentration decreases over time as it is consumed within the modeled reaction framework.
- Hydrogen and carbon dioxide concentrations increase with time, reflecting product formation within the system.
- Oxygen concentration remains negligible, indicating limited transport from the cathode side.

The simulation illustrates interactions between transport behavior and concentration gradients under simplified assumptions, providing qualitative insight relevant to system design considerations. COMSOL is used to interpret how spatial nonuniformities and concentration behavior can support or constrain the comparative trends identified in MATLAB within the same operating envelope. A reproducible workflow for the COMSOL Multiphysics setup, boundary assignments, solver execution, and post-processing steps is provided in Appendix A.

### 3.4. MINITAB for DoE analysis

A Design of Experiment (DoE) was setup in MINITAB for statistically comparing anode materials. The goal was to quantify the effect of electrode material type on MFC output voltage and to statistically find the best option among the five considered: platinum, graphene, graphite felt, carbon nanotube, and stainless steel. A voltage dataset for each material was created by taking a small number of experimentally observed values and then extrapolating these by generating additional values with higher coverage but while still conserving the experimentally observed trend. This created dataset was based on the prototype ranges and on the trend behavior across the modeling layers and was used for exploring whether comparative differences still remain statistically separable under the stated assumptions. The response variable was the mean voltage (V) measured under equivalent substrate and load conditions.

#### 3.4.1. Testing of assumptions.

**Fig 10:**
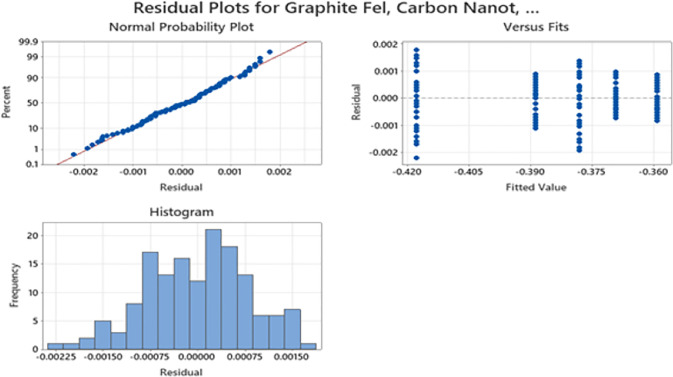
Residual plots for validation of assumptions.

The dataset is first tested for certain assumptions including normality and equal variances across different electrode materials. It is accomplished using residual plots analysis in MINITAB, and the resulting residual plots are shown in Fig 10. The residual plots indicate no major violations of normality or equal-variance assumptions, supporting the use of ANOVA as a comparative screening tool for the constructed dataset.

#### 3.4.2. ANOVA and Statistical Significance.

**Fig 11:**
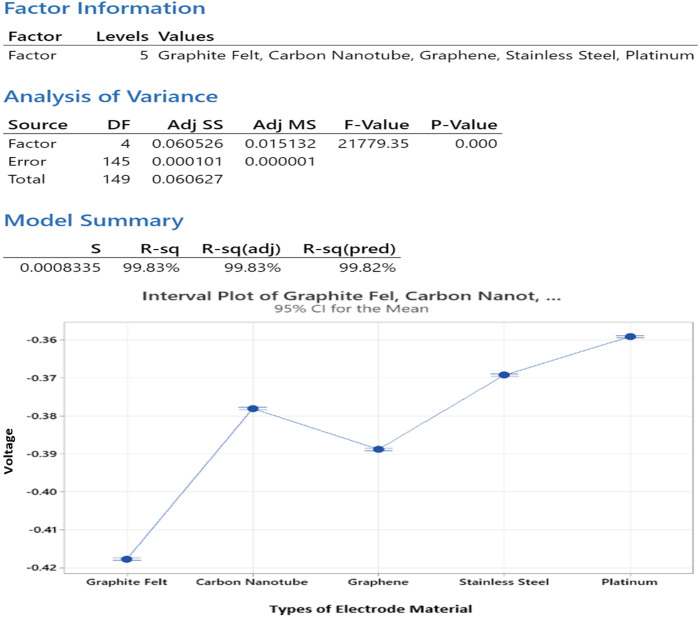
ANOVA Display and Interval Plot for Electrode Material using MINITAB.

The ANOVA showed that electrode types produced a statistically significant difference (p < 0.05). The results demonstrate that selection of anode material produces measurable differences in MFC performance. Fig 11 displays the ANOVA summary table which includes between-group and within-group sum of squares together with mean squares, F-value, and p-value information. The high F-ratio demonstrates a powerful influence of material type on voltage output.

#### 3.4.3. Post-hoc Fisher Analysis.

**Fig 12:**
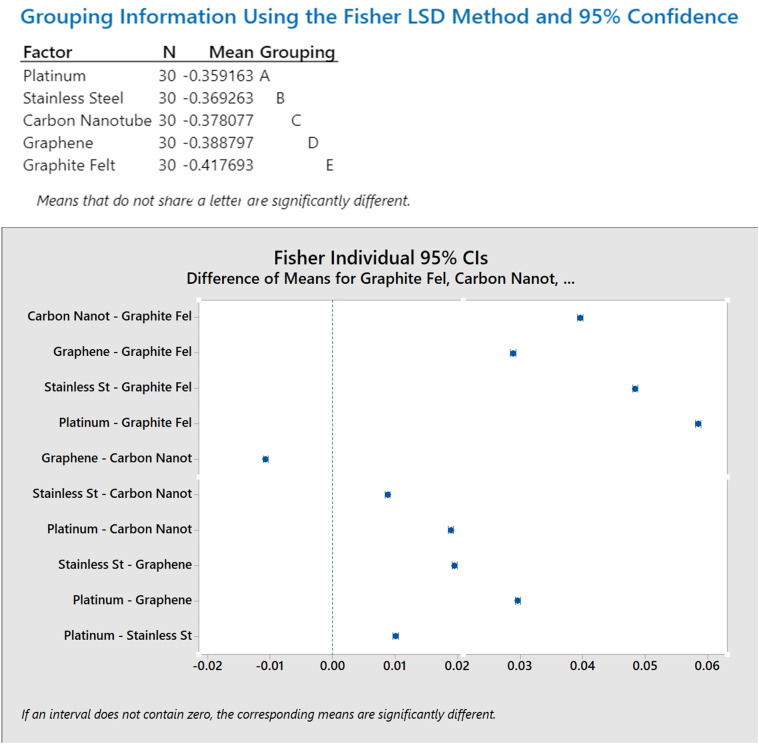
Post-hoc Fisher LSD Analysis using MINITAB.

A Fisher’s Least Significant Difference (LSD) test was performed to determine the specific materials that differ from one another. Fig 12 displays the Fisher LSD 95% confidence intervals for the pairwise differences in mean voltage between electrode materials.

The key findings are listed as:

- The highest voltage production was observed for the Platinum electrode, which was statistically different from all other materials.
- Stainless Steel, Carbon Nanotube, and Graphene exhibited intermediate voltage outputs, with each material being statistically distinct from the others.
- Graphite Felt produced the lowest voltage output and was statistically different from all other electrode materials.

The findings offer statistically supported guidance for choosing the best electrode materials in subsequent design versions. A reproducible workflow for the MINITAB statistical analysis, including the one-way ANOVA and Fisher’s post-hoc comparisons, is provided in Appendix A.

### 3.5. Summary of Insights

The application of the proposed MFC framework was investigated with an integrated suite of simulations, where limited observations from prototype testing were used to support the grounding of voltage ranges and to inform a comparative screening exercise, but not to be used directly as a validation dataset in its entirety. Multidimensional characterization of system behavior was made possible through the simultaneous consideration of thermodynamic/kinetic, spatial and statistical aspects in four simulation environments: DWSIM, MATLAB, COMSOL and MINITAB.

The key findings are summarized below:

- **Thermodynamic feasibility and mass–energy consistency (DWSIM):**

DWSIM was employed to determine a steady-state thermodynamic reference envelope as well as to check the consistency of mass and energy balances between the anode, PEM and cathode with respect to the stated conversion assumptions. In this system level representation, the PEM block is incorporated to account for selective proton transport with limited back-mixing, which generates bounded outlet-composition and energy trends to aid interpretation of feasible operating windows.

- **Electrochemical Kinetics (MATLAB):**

Simulations using the Butler-Volmer equation showed that Platinum and Graphene exhibited the most promising behavior as anode materials due to their low overpotential and high current densities. Stable microbial growth as well as substrate breakdown was shown using Monod kinetics.

- **Biofilm and Species Transport (COMSOL):**

The observed biofilm growth near the anode and the estimated concentration gradients of glucose, H ⁺ ion, CO₂ and O₂ were reproduced in the spatial model. The study results show that the design of the anode chamber and separator interface work well.

- **Statistical Evaluation (MINITAB):**

ANOVA results on the data suggest the voltages between the materials are statistically different from one another. A Fisher LSD test proved the two most likely candidates to be used as anode materials were Platinum and Graphene.

## 4. Economic, environmental and societal benefits

MFCs can offer a combined set of benefits that conventional wastewater treatment and standalone renewable energy systems typically deliver separately. With increasing emphasis on sustainable infrastructure and circular economy thinking, MFCs are often discussed as a technology that can contribute to both resource recovery and environmental protection within a single treatment platform.

### 4.1. Economic benefits

In some wastewater treatment configurations, MFCs may reduce operating burdens tied to energy use and sludge handling. Compared to conventional systems, which often require aeration of the tank contents (energy-intensive), MFCs can work without mechanical aerators, and can generate electricity during the treatment process, thus potentially providing a portion of the energy required for the process under the right conditions. Fewer moving parts can result in less maintenance, and the use of naturally-occurring bacteria and passive electron transfer may eliminate the need for more complex systems and specialized additives. Although platinum gives the best results, cheaper carbon-based electrodes can also be used. The modular design allows for phased scale up, although the overall impact on cost is dependent on local conditions, scale and implementation choices.

### 4.2. Environmental benefits

MFCs support environmental sustainability by reducing pollutant loads and, in some cases, lowering greenhouse gas emissions when recovered electricity displaces grid or on-site fossil-derived power. Under favorable conditions, MFC-based systems may also limit methane generation relative to certain anaerobic treatment routes, supporting a lower carbon footprint. Compared with activated sludge processes, MFCs may produce less biological sludge, which can reduce downstream treatment and disposal burdens. In addition to lowering chemical oxygen demand, MFCs can be positioned to support removal of nitrogen and phosphates when paired with appropriate downstream processes.

### 4.3. Societal benefits

MFCs have potential to improve sanitation access and public health, particularly in underserved settings. Their modular design allows deployment in remote or off-grid locations where centralized infrastructure is unavailable or cost-prohibitive, including small community-scale installations. By treating wastewater closer to the source and reducing pathogen loads, MFC-based treatment may lower waterborne disease risks and support safer reuse of treated water for applications such as agriculture or aquaculture, subject to local standards and any required post-treatment.

## 5. Conclusions, future recommendations and limitations

This study presents a reproducible, multi-platform workflow for early-stage interpretation and comparative screening of MFC design choices for wastewater treatment with energy recovery. Under stated assumptions, DWSIM was used to provide steady-state thermodynamic bounding and mass–energy consistency checks across the anode, PEM, and cathode, while MATLAB was used to generate comparative Butler–Volmer and Monod-based trends for anode-material sensitivity. COMSOL was applied as a spatial interpretation layer to visualize concentration gradients and biofilm-related distributions in a simplified anode domain, supporting qualitative assessment of transport behavior near the electrode interface. MINITAB was used for comparative statistical screening using a constructed voltage dataset guided by exploratory prototype observations, and the analysis indicates statistically distinguishable voltage differences across anode materials, with platinum producing the highest mean voltage and platinum and graphene emerging as top candidates in post-hoc comparisons.

The findings should be interpreted within the limits of the modeling assumptions and the intended screening purpose. The simulations were conducted under idealized conditions and used synthetic substrates, and the statistical screening relied on a constructed dataset informed by limited prototype voltage ranges rather than a fully validation-grade experimental campaign. In addition, the COMSOL model is a two-dimensional representation with a static biofilm layer intended for qualitative transport insight rather than fully predictive biological kinetics, and the system-level analyses are designed to bound feasible behavior rather than replace calibrated mechanistic validation. Future work should therefore prioritize benchmarking the workflow against expanded laboratory datasets and literature-reported performance under comparable operating conditions, followed by trials using municipal or industrial wastewater to assess robustness under realistic variability. Further extensions should examine lower-cost electrode alternatives to platinum-type materials, incorporate long-term operation effects such as biofouling and durability, and evaluate scale-up and stacking behavior under different electrical configurations and load conditions for decentralized treatment applications.

## Supporting information

S1 Text Algorithms for Simulations. It contains simulation algorithms to model MFCs in various softwares.
(DOCX)

S1 File Appendix A. (DOCX)

## Abbreviations

ANOVA: Analysis of Variance
DoE: Design of Experiment
LSD: Least Significant Difference
MFC: Microbial Fuel Cell
PEM: Proton Exchange Membrane
