# Supplementary material for: Microbial fuel cells for wastewater treatment and electricity production: A multi-platform simulation workflow
Source: PLoS One. 2026 May 7;21(5):e0348078. doi: 10.1371/journal.pone.0348078 (PMC13152150; doi:10.1371/journal.pone.0348078)
Supplement: S1 Text — It contains simulation algorithms to model MFCs in various softwares. (DOCX) [file pone.0348078.s001.docx]

**Algorithms for Simulation Softwares**

# S1- DWSIM

**S1.1 Software and settings**

- **Software:** DWSIM (steady-state simulation)
- **Thermodynamics:** Peng–Robinson (PR) property package
- **Component set:** H₂O, H₂, CO₂, O₂, C₆H₁₂O₆ (glucose)

**S1.2 Flowsheet construction**

**S1.2.1 Unit operations**

Create the following blocks:

1. **RGIBBS-1** (Gibbs reactor)
2. **SEP-1** (component splitter / separator)
3. **MIX-1** (mixer)
4. **R-1** (stoichiometric or conversion reactor; cathode reaction)

**S1.2.2 Material streams**

Create and label the following streams:

- Feed
- Anode outlet 1
- Anode outlet 2
- Separated hydrogen ions
- Carbon dioxide and unreacted products
- Oxygen supply
- Mix outlet
- Water
- Unreacted products

**S1.2.3 Connections**

Connect the blocks and streams in the following order:

1. Feed → RGIBBS-1
2. Configure RGIBBS-1 to generate two outlets and label them:
   - Anode outlet 1
   - Anode outlet 2
3. Anode outlet 1 → SEP-1
4. Configure SEP-1 to generate two outlets and label them:
   - Separated hydrogen ions
   - Carbon dioxide and unreacted products
5. Separated hydrogen ions → MIX-1
6. Oxygen supply → MIX-1
7. Label the MIX-1 outlet stream as mix outlet
8. Mix outlet → R-1
9. Configure R-1 to generate two outlets and label them:
   - Water
   - Unreacted products

**S1.3 Feed stream specification**

Define stream **feed** using the following conditions:

**S1.3.1 State conditions**

- Temperature: **298.15 K**
- Pressure: **101325 Pa**

**S1.3.2 Flow specification**

- Mass flow: **0.659581 kg·s⁻¹**
- Molar flow: **13.5398 mol·s⁻¹**
- Volumetric flow: **0.113124 m³·s⁻¹**

**S1.3.3 Overall composition (mole fraction)**

- H₂O = **0.4444444**
- H₂ = **0.3333333**
- C₆H₁₂O₆ = **0.2222222**
- O₂ = **0**
- CO₂ = **0**

**S1.4 RGIBBS reactor configuration (RGIBBS-1)**

1. Set RGIBBS-1 to operate at the inlet stream temperature and pressure (298.15 K, 101325 Pa).
2. Select Gibbs free-energy minimization for the component set (H₂O, H₂, CO₂, O₂, C₆H₁₂O₆).
3. Enable phase handling such that **two outlet streams** are produced (vapor and liquid outlets).
4. Run the simulation.

**S1.5 Separator configuration (SEP-1)**

Configure SEP-1 as a component splitter using the following component-wise split fractions:

| **Component** | **To “separated hydrogen ions”** | **To “carbon dioxide and unreacted products”** |
| --- | --- | --- |
| H₂ | 1.0 | 0.0 |
| H₂O | 0.0 | 1.0 |
| CO₂ | 0.0 | 1.0 |
| O₂ | 0.0 | 1.0 |
| C₆H₁₂O₆ | 0.0 | 1.0 |

Run the simulation.

**S1.6 Oxygen supply and cathode reactor configuration**

**S1.6.1 Oxygen supply stream**

Define **oxygen supply** as:

- Composition: O₂ mole fraction = **1.0**
- Temperature: **298.15 K**
- Pressure: **101325 Pa**

Set the O₂ molar flow based on the molar flow of H₂ in **separated hydrogen ions**:

- **n(O₂) = 0.5 × n(H₂)**

**S1.6.2 Cathode reaction block (R-1)**

Define the following reaction in R-1:

- **H₂ + 0.5 O₂ → H₂O**

Set conversion to consume H₂ (complete conversion if O₂ is stoichiometric or in excess). Run the simulation.

**S1.7 Outputs for export**

Export stream property reports (state and composition) for:

- Feed
- Anode outlet 1
- Anode outlet 2
- Separated hydrogen ions
- Carbon dioxide and unreacted products
- Water
- Unreacted products

# S2- MATLAB

**S2.1 Software environment**

- **Software:** MATLAB
- Ensure the working directory contains the scripts required to execute the two simulations described below.

**S2.2 Anode potential simulation procedure**

**S2.2.1 Workspace initialization**

Clear workspace, close figures, and clear command window:

clear;

close all;

clc;

**S2.2.2 Constant definitions**

Define the following constants:

- Faraday constant: F = 96485; (C/mol)
- Gas constant: R = 8.314; (J/mol·K)
- Temperature: T = 298; (K)
- Electrons transferred: n = 8;
- Charge transfer coefficient: alpha = 0.5;

**S2.2.3 Material set and exchange current densities**

1. Define the anode material labels:

material = {'Graphite Felt','Carbon Nanotube','Graphene','Stainless Steel','Platinum'};

1. Define exchange current densities corresponding to the material order:

i0_values = [1e-6, 1e-4, 1e-3, 1e-7, 1e-2];

**S2.2.4 Current density range**

Define a logarithmically spaced current density vector (A/m²):

i = logspace(-7,-1,100);

**S2.2.5 Overpotential and anode potential calculations**

1. Create a figure and enable multiple curves:

figure;

hold on;

1. For each material index k = 1:length(material):

- Assign: i0 = i0_values(k);
- Compute anode overpotential:
  eta = (R*T/(alpha*n*F)) * log(i./i0);
- Compute anode potential using a fixed equilibrium potential:
  E_anode = 0.3 - eta;
- Plot curve:
  plot(i, E_anode, 'LineWidth', 2, 'DisplayName', material{k});

**S2.2.6 Plot formatting**

1. Set x-axis scale to logarithmic:
   set(gca,'XScale','log');
2. Apply labels and title:

- xlabel('Current Density (A/m^2)');
- ylabel('Anode Potential (V)');
- title('Effect of Anode Material on Anode Potential');

1. Display legend and finish plot:

- legend;
- hold off;

**S2.3 Microbial growth simulation procedure**

**S2.3.1 Workspace initialization**

Clear workspace, close figures, and clear command window:

clear;

close all;

clc;

**S2.3.2 Initial conditions and time span**

1. Define the initial state vector:
   Y0 = [10; 0.1];
   where Y(1) = S (substrate) and Y(2) = X (biomass)
2. Define the simulation time interval (hours):
   tspan = [0 100];

**S2.3.3 ODE solution**

Solve the ODE system using ode45:

[t, Y] = ode45(@mfc_ode, tspan, Y0);

**S2.3.4 Plot generation**

1. Plot substrate and biomass versus time:

plot(t, Y(:,1), 'r', t, Y(:,2), 'g');

1. Add legend, labels, and title:

- legend('Substrate', 'Biomass');
- xlabel('Time (h)');
- ylabel('Concentration');
- title('Substrate and Biomass Concentration over time');

1. Enable grid and set y-axis limits:

- grid on;
- ylim([-10 10]);

**S2.3.5 ODE function specification**

Define the ODE function:

1. Function header:
   function dYdt = mfc_ode(t, Y)
2. Parameter definitions:

- k_s = 0.2;
- K_s = 1.0;
- mu_max = 0.1;

1. State variable mapping:

- S = Y(1);
- X = Y(2);

1. Derivative equations:

- Biomass growth:
  dXdt = mu_max * (S/(K_s + S)) * X;
- Substrate consumption:
  dSdt = -k_s * (S/(K_s + S)) * X;

1. Return derivative vector and end function:

- dYdt = [dSdt; dXdt];
- end

**S2.4 Execution procedure**

1. Run the anode potential simulation procedure (Section S2.2) to generate the anode potential plot.
2. Run the microbial growth simulation procedure (Section S2.3) to generate the time-series concentration plot.

# S3- COMSOL Multiphysics

**S3.1 Software environment**

- **Software:** COMSOL Multiphysics (2D model)
- **Physics interface:** Transport of Diluted Species (time-dependent)

**S3.2 Model scope and assumptions**

- Construct a simplified 2D anode-domain model to examine spatial transport behaviour and relative concentration-gradient trends under representative conditions.
- Represent the biofilm as a static layer adjacent to the anode-side boundary, used to impose spatially varying transport and reaction activity (no dynamic biofilm growth kinetics).

**S3.3 Geometry definition**

1. Create a 2D rectangular domain representing the anode compartment cross-section.
2. Set the x-direction length to:
   - L = 1 mm (x from 0 to 1 mm)
3. Set the y-direction height to a representative value (consistent unit scale), e.g.:
   - H = 1 mm (y from 0 to 1 mm)

**S3.4 Dependent variables and species list**

Define diluted-species concentration fields (units: mol/m³) for the species tracked in the time-evolution plot:

- Glucose: **c_C6H12O6_**
- Water: **c_H2O_**
- Carbon dioxide: **c_CO2_**
- Hydrogen: **c_H2_**
- Oxygen: **c_O2_**

**S3.5 Parameter definitions**

**S3.5.1 Diffusion coefficients (aqueous, representative values)**

Assign diffusion coefficients (m²/s):

- D_C6H12O6_ = 6.0×10⁻¹⁰
- D_H2O_ = 2.3×10⁻⁹
- D_CO2_ = 1.9×10⁻⁹
- D_H2_ = 4.5×10⁻⁹
- D_O2_ = 2.0×10⁻⁹

**S3.5.2 Biofilm activity scaling and kinetic constant**

Define:

- **L = 1e-3 m** (domain length in meters)
- **k = 1.0×10⁻⁵ s⁻¹** (representative first-order rate constant)

**S3.6 Biofilm representation (static spatial layer/activity profile)**

1. Define an analytic function for a spatial biofilm/activity indicator that decreases linearly across x:

**b(x) = 1 − (x / L)**

1. Implement b(x) as either:
   - a variable defined in *Definitions → Variables*, or
   - an analytic function defined in *Definitions → Functions*
2. Use b(x) as a multiplier for reaction intensity (Section S3.8) to reproduce a spatially varying biofilm influence across the domain.

**S3.7 Physics setup: Transport of Diluted Species**

1. Add Transport of Diluted Species interface.
2. Add the five species from Section S3.4.
3. For each species, assign its diffusion coefficient from Section S3.5.1.

**S3.8 Reaction-source terms (representative stoichiometric framework)**

Define a glucose-consumption rate that is spatially modulated by the biofilm indicator:

**R = k · b(x) · c_C6H12O6_** (mol/m³·s)

Apply reaction/source terms to each species in the domain using a representative glucose-to-products stoichiometry:

- Glucose consumption:
  - **R_C6H12O6_ = −R**
- Water consumption:
  - **R_H2O_ = −6R**
- CO₂ production:
  - **R_CO2_ = +6R**
- H₂ production:
  - **R_H2_ = +12R**
- Oxygen:
  - **R_O2_ = 0**

Implement these under the Reactions (or equivalent source/reaction term settings) of the diluted-species interface.

**S3.9 Initial conditions**

Set initial concentrations (mol/m³) uniformly across the domain:

- c_C6H12O6_(0) = 0.30
- c_H2O_(0) = 0.35
- c_CO2_(0) = 0.70
- c_H2_(0) = 1.45
- c_O2_(0) = 0.00

**S3.10 Boundary conditions**

1. Apply no-flux (insulation) boundaries for all species on the rectangle boundaries as the default transport condition.
2. To keep oxygen transport limited, retain:
   - c_O2 initialized at 0, and
   - no-flux boundaries (oxygen remains negligible without an imposed inlet).

**S3.11 Meshing strategy**

1. Generate a physics-controlled mesh (normal or finer).
2. Apply local mesh refinement near the anode-side boundary (x = 0) if needed to resolve sharper gradients in the region adjacent to the biofilm activity zone.

**S3.12 Study and solver configuration**

1. Select Time Dependent study.
2. Set the simulation time range:
   - t = 0 to 3600 s
3. Use automatic time stepping with a maximum step size small enough to resolve early-time transients (e.g., ≤ 10–50 s).

**S3.13 Post-processing instructions**

**S3.13.1 Biofilm spatial profile plot**

1. Create a Cut Line 2D from x = 0 to x = 1 mm at a fixed y (e.g., y = 0.5 mm).
2. Plot b(x) along the cut line to obtain the spatial biofilm/activity distribution.

**S3.13.2 Species concentration vs time**

1. Define a Point Evaluation location within the domain (e.g., x = 0.5 mm, y = 0.5 mm).
2. Create a Global/Point plot of the following concentrations versus time:
   - c_C6H12O6_, c_H2O_, c_CO2_, c_H2_, c_O2_

# S4- MINITAB

**S4.1 Software environment**

- **Software:** MINITAB (desktop)
- **Analysis module:** Stat → ANOVA → One-Way…

**S4.2 Data structure and worksheet preparation**

**S4.2.1 Worksheet format**

1. Create/open a MINITAB worksheet.
2. Arrange the response values such that each material is a separate column (one column per factor level).
3. Use five columns labeled as follows:
   - C1: Graphite Felt
   - C2: Carbon Nanotube
   - C3: Graphene
   - C4: Stainless Steel
   - C5: Platinum

**S4.2.2 Data validation**

1. Confirm each column contains the voltage values for the corresponding material.
2. Confirm all entries are numeric and there are no blank cells within the range.

**S4.3 One-Way ANOVA procedure**

**S4.3.1 Open One-Way ANOVA dialog**

1. Navigate to: Stat → ANOVA → One-Way
2. In the drop-down at the top of the dialog, select:
   **Response data are in a separate column for each factor level**

**S4.3.2 Specify response columns**

1. Under Responses, select and enter the five columns:
   - Graphite Felt
   - Carbon Nanotube
   - Graphene
   - Stainless Steel
   - Platinum

**S4.4 Options configuration**

**S4.4.1 Variance assumption and confidence settings**

1. Click Options
2. Ensure “Assume equal variances” is checked.
3. Set Confidence level to 95.
4. Set Type of confidence interval to Two-sided.
5. Click OK.

**S4.5 Fisher–LSD multiple comparisons configuration**

**S4.5.1 Select comparison method and error rate**

1. Click Comparisons
2. Set Error rate for comparisons to 5.
3. Under “Comparison procedures assuming equal variances”, select **Fisher**

**S4.5.2 Select comparison outputs**

1. Under Results, enable:
   - Interval plot for differences of means
   - Grouping information
   - Tests
2. Click OK.

**S4.6 Graph generation configuration**

**S4.6.1 Data plots**

1. Click Graphs
2. Under Data plots, enable:
   - Interval plot
   - Individual value plot
   - Boxplot of data

**S4.6.2 Residual plots**

1. Under Residual plots, select: Three in one
2. Click OK.

**S4.7 Results table configuration**

**S4.7.1 Output table settings**

1. Click Results…
2. Set Display of results to:
   - Expanded tables
3. Ensure the following are enabled:
   - Method
   - Factor information
   - Analysis of variance
   - Model summary
   - Means
4. Click OK.
