# Supplementary material for: Microbial fuel cells for wastewater treatment and electricity production: A multi-platform simulation workflow
Source: PLoS One. 2026 May 7;21(5):e0348078. doi: 10.1371/journal.pone.0348078 (PMC13152150; doi:10.1371/journal.pone.0348078)
Supplement: S1 File — (DOCX) [file pone.0348078.s002.docx]

# Appendix A

**Algorithm A1. DWSIM steady-state flowsheet (anode–separator–cathode)**

**Inputs:** Peng Robinson; components {H₂O, H₂, CO₂, O₂, C₆H₁₂O₆}; feed composition and conditions
**Outputs:** Converged stream tables; mass/energy balance summaries

**Procedure**

1. **Initialize:** Create new case → select **Peng–Robinson** → add components {H₂O, H₂, CO₂, O₂, C₆H₁₂O₆}.
2. **Define Feed stream:** T = 298.15 K; P = 101325 Pa; total flow = 1 mol/s; z(C₆H₁₂O₆)=0.1, z(H₂O)=0.9, others = 0.
3. **Build flowsheet units:** Place **RGIBBS** (anode proxy), **Separator/Splitter** (PEM proxy), **Stoichiometric reactor** (cathode proxy), and required connectors (mixer if needed).
4. **Configure RGIBBS (anode proxy):** Connect Feed → RGIBBS; set operating T,P to feed conditions; solve for equilibrium.
5. **Configure Separator (PEM proxy):** Split RGIBBS outlet into two streams:
   - Stream A: passes **H₂ only** (split fraction H₂=1; all other components=0)
   - Stream B: passes **all non-H₂ species** (split fraction for H₂=0; others=1)
6. **Configure cathode feed:** Create pure O₂ stream at 298.15 K and 101325 Pa; set molar flow to satisfy stoichiometry with Stream A: n(O₂)=0.5·n(H₂).
7. **Configure stoichiometric reactor (cathode proxy):** Reaction: H₂ + 0.5O₂ → H₂O; set conversion to consume H₂ (with stoichiometric or excess O₂).
8. **Run & export:** Solve flowsheet; export stream compositions/flows and unit mass/energy balances.

**Algorithm A2. MATLAB anode potential comparison (Butler–Volmer form)**

**Inputs:** Material list; i₀ values; constants {F, R, T, n, α}; current-density range
**Outputs:** E–i curves for all materials

**Procedure**

1. Initialize MATLAB session (clear workspace/figures).
2. Set constants: F=96485; R=8.314; T=298; n=8; α=0.5.
3. Define materials M = {Graphite Felt, CNT, Graphene, Stainless Steel, Platinum}.
4. Define exchange current densities i₀(M) = {1e−6, 1e−4, 1e−3, 1e−7, 1e−2}.
5. Generate current density vector i = logspace(−7, −1, 100).
6. For each material m in M:
   6.1 Compute overpotential η(m,i) = (R·T/(α·n·F))·ln(i/i₀(m)).
   6.2 Compute anode potential E(m,i) using the chosen baseline convention (E = E₀ + η, with fixed E₀).
   6.3 Plot E(m,i) vs i (log x-axis) with a unique label.
7. Add axis labels, legend; export the figure.

**Algorithm A3. MATLAB microbial growth (Monod ODE, time domain)**

**Inputs:** Initial states S₀, X₀; parameters μ_max_, K_s_, k_s_; time span
**Outputs:** Time profiles S(t), X(t)

**Procedure**

1. Initialize MATLAB session (clear workspace/figures).
2. Set initial state: Y₀ = [S₀; X₀] = [10; 0.1].
3. Set time span: t ∈ [0, 100] hours.
4. Set parameters: μ_max_=0.1; K_s_=1.0; k_s_=0.2.
5. Define ODE system for Y=[S;X]:
   - dX/dt = μ_max_·(S/(K_s_+S))·X
   - dS/dt = −k_s_·(S/(K_s_+S))·X
6. Solve with an adaptive integrator (e.g., ode45).
7. Plot S(t) and X(t); label axes and export the figure.

**Algorithm A4. COMSOL 2D Transport of Diluted Species (time dependent)**

**Inputs:** 2D rectangle geometry; diffusion coefficients; initial conditions; boundary constraints; reaction term with spatial activity b(x)
**Outputs:** Concentration fields, line profiles, time traces

**Procedure**

1. Create **2D model** → add physics **Transport of Diluted Species (time dependent)**.
2. Define species: c_C₆H₁₂O₆_, c_H2O_, c_CO2_, c_H2_, c_O2_.
3. Define geometry: rectangle with dimensions 1 mm × 1 mm.
4. Define parameters: diffusion coefficients for each species; kinetic constant k.
5. Define spatial activity function: b(x) = 1 − x/L, with L = 1 mm.
6. Set initial concentrations:
   c_C₆H₁₂O₆_=1; c_H2O_=55.5; c_CO2_=0; c_H2_=0; c_O2_=0.01 (mol/m³).
7. Apply boundary constraints for oxygen: set c_O2_ at x=0 to 0.01 and at x=L to 0 (mol/m³).
8. Define reaction rate: R = k·b(x)·c_glucose.
9. Apply source/sink terms consistent with the selected stoichiometric mapping: glucose consumed at rate R; products generated proportionally (CO₂, H₂, H₂O) per the defined coefficients.
10. Solve time-dependent study over the specified horizon.
11. Export: 2D concentration maps, a representative cut-line profile, and point/time traces.

**Algorithm A5. MINITAB one-way ANOVA with Fisher’s LSD (separate columns)**

**Inputs:** Worksheet with five response columns (one per material); α=0.05
**Outputs:** ANOVA table; Fisher–LSD tests/grouping; plots (interval, individual, boxplot, residuals)

**Procedure**

1. Create/open worksheet; place replicate response values into columns:
   C1 Graphite Felt; C2 Carbon Nanotube; C3 Graphene; C4 Stainless Steel; C5 Platinum.
2. Open: **Stat → ANOVA → One-Way**.
3. Select data format: **Response data are in a separate column for each factor level**.
4. Responses = {C1, C2, C3, C4, C5}.
5. Options: enable **Assume equal variances**; set **95% two-sided** intervals.
6. Comparisons: set error rate **5**; choose **Fisher**; enable **Tests**, **Grouping information**, **Interval plot for differences of means**.
7. Graphs: enable **Interval plot**, **Individual value plot**, **Boxplot**; residuals = **Three in one**.

Results: **Expanded tables** with Method, Factor information, ANOVA, Model summary, Means.
